# Editorial: Innovative strategies for enhancing plant resilience to phytopathogenic microbes

**DOI:** 10.3389/fpls.2025.1632709

**Published:** 2025-06-10

**Authors:** Temoor Ahmed, Dirk Janssen, Yasser Nehela

**Affiliations:** ^1^ Xianghu Laboratory, Hangzhou, China; ^2^ Instituto Andaluz de Investigación y Formación Agraria (IFAPA) Centro La Mojonera, Almería, Spain; ^3^ Department of Agricultural Botany, Faculty of Agriculture, Tanta University, Tanta, Egypt

**Keywords:** phytopathogens, plant resilience, disease management, host-pathogen interactions, genetic engineering, biocontrol agents, sustainable agriculture

Plant diseases pose a significant threat to global food security, biodiversity, and economic stability. Plants face constant attack from a diverse array of pathogens including bacteria, fungi, viruses, and nematodes that collectively cause devastating agricultural losses (Ristaino et al., 2021; Wang et al., 2022). The financial impact is staggering, with annual crop yield losses from pathogens and pests estimated at $220 billion worldwide. These losses don’t merely represent economic figures; they translate to real food shortages, compromised regional economies, and cascading socioeconomic challenges (Singh et al., 2023). The extensive use of traditional chemical pesticides and antibiotics involving toxic substances has become more challenging due to severe ecotoxicological challenges (Ahmed et al., 2023). Thus, there is an urgent need to develop effective and robust eco-friendly plant disease management approaches to overcome global food crisis.

A recent Research Topic of “Frontiers in Plant Science” explores groundbreaking research aimed to enhancing plant resilience to phytopathogenic microbes. The Research Topic features nine original research papers and one comprehensive review that collectively advances our understanding of sustainable agriculture through enhanced disease resistance. The featured research illuminates several critical areas: the molecular interactions between plants and pathogens, novel approaches to disease management, biotechnological innovations for improved resistance, and the complex interplay between climate change and disease dynamics. For example, Ayaz et al. showed that *Bacillus subtilis* BS-2301 exhibits strong antifungal activity against *Sclerotinia sclerotiorum* via ROS accumulation, OA reduction, and hyphal damage. It also promotes plant growth, enhances antioxidant defense, and upregulates disease-resistant genes. Overall, *Bacillus subtilis* BS-2301 is a promising biocontrol agent for sustainable agriculture with broad-spectrum antagonism and growth-promoting traits. In another study, Arbuscular mycorrhizal fungi (AMF) *Rhizophagus intraradices* enhance *Lycium barbarum* resistance to *Fusarium solani* by boosting phenylpropane metabolism, increasing lignin (141.65%) and flavonoids (44.61%), and elevating defense-related enzymes and hormones. AMF symbiosis improves plant growth (24.83% height increase) and strengthens early pathogen response, offering a sustainable biocontrol strategy against root rot (Li et al.).

Chancellor et al. revealed that *Gaeumannomyces hyphopodioides* controls take-all disease by locally modifying wheat root gene expression, suppressing cell wall–related genes (*CESA*, *XTH*), and forming lipid-rich subepidermal vesicles (SEVs). These findings highlight a novel biocontrol mechanism, offering potential strategies for enhancing wheat resistance against *G. tritici* in the absence of resistant cultivars. Another study identifies PeVn1, a novel protein elicitor from *Verticillium nonalfalfae*, which triggers plant immune responses via NbBAK1/NbSOBIR1-dependent cell death, ROS burst, and defense activation. PeVn1 enhances resistance against multiple pathogens, offering potential for developing protein-based biocontrol agents in sustainable agriculture (Zhang et al.). Chen et al. demonstrated that LAZ1 and LAZ1H1 are evolutionarily conserved positive regulators of SAR, modulating *CBP60g* and *SARD1* expression and SA/NHP biosynthesis. Their overexpression enhances pathogen resistance, highlighting their potential as targets for improving plant immunity in crops.

The application of 0.3% tetramycin and reduced-dose tebuconazole·azoxystrobin synergistically controls Taizishen leaf diseases (90.03–90.46% efficacy), enhances physiological activity (electrical signals, photosynthesis, nutrient transport), improves growth/quality metrics, and reduces pesticide use, offering an efficient and sustainable disease management strategy (Tian et al.). Ahmed et al. demonstrated that *Luteibacter pinisoli* DP2–30 effectively combats pine wilt disease through potent nematicidal activity (>95% mortality), suppressed egg hatching (43-49%), and microbiome modulation—notably enriching Rhodanobacteraceae. Its dual-action mechanism (direct pathogen suppression and host microbiota restructuring) offers promising eco-friendly PWD control. Another recent study identified phage cocktails (BPC-1) with exceptional bacterial wilt control efficacy (99.25%), combining broad-host-range (YL1/YL4) and high-efficacy (YL2/YL3) phages. Structural analyses of tail fiber proteins reveal key amino acid determinants for host specificity, offering optimized phage-based solutions for sustainable bacterial wilt management (He et al.). Yang et al. revealed that *P. polysora* infection restructures maize endophytic communities, reducing diversity and network complexity while revealing key fungal associations (e.g., *Alternaria*-resistance correlation). Temperature-driven microbial assembly and differential regional resilience offer new insights for developing microbiome-based SCR management strategies. Last, a review published by Masood et al. demonstrated that nano-enabled immunomodulation offers a sustainable approach to enhance plant disease resistance through engineered nanomaterials that trigger immune responses, deliver bioactive compounds, and reshape microbiomes. Additionally, this review systematically examines current advances in nano-enabled immunomodulation approaches, explores their underlying mechanisms, and identifies key research directions to address current limitations for eco-friendly plant disease control. Together, these studies provide a foundation for developing more resilient agricultural systems capable of withstanding emerging pathogenic threats.
